# Modulations in gastrointestinal microbiota during postpartum period fulfill energy requirements and maintain health of lactating Tibetan cattle

**DOI:** 10.3389/fmicb.2024.1369173

**Published:** 2024-08-20

**Authors:** Jing Liu, Anum Ali Ahmad, Chen Yang, Jianbo Zhang, Juanshan Zheng, Zeyi Liang, Fang Wang, Huan Zhai, Shuanghong Qin, Feng Yang, Xuezhi Ding

**Affiliations:** ^1^Key Laboratory of Yak Breeding Engineering, Lanzhou Institute of Husbandry and Pharmaceutical Sciences, Chinese Academy of Academy of Agricultural Sciences, Lanzhou, China; ^2^The Roslin Institute and Royal (Dick) School of Veterinary Studies, University of Edinburgh, Edinburgh, United Kingdom; ^3^Plateau Livestock Genetic Resources Protection and Innovative Utilization Key Laboratory of Qinghai Province, Key Laboratory of Animal Genetics and Breeding on Tibetan Plateau, Ministry of Agriculture and Rural Affairs, Qinghai Academy of Animal and Veterinary Medicine, Qinghai University, Xining, China; ^4^Department of Endocrinology, The Second People's Hospital of Gansu Province, Lanzhou, Gansu, China

**Keywords:** gut microbiota, Tibetan cattle, postpartum period, lactating cattle, health

## Abstract

**Introduction:**

Postpartum period of dairy cattle is an important phase of their life mainly associated with the changes in physiology, rumen function, and energy metabolism. Studies have shown that gut microbial composition undergoes drastic changes during the postpartum period. However, little is known about the temporal variations in digestive tract microbiota in postpartum Tibetan cattle. The aim of this study was to investigate the temporal variations in blood metabolites, ruminal fermentation, and microbial community of oral, rumen, and gut in lactating Tibetan cattle during postpartum.

**Methods:**

We collected blood, saliva, rumen fluid, and fecal samples from lactating Tibetan cattle during 1st week (1 W), the 2nd week (2 W), the 1st month (1 M), and the 2nd month (2 M) of the postpartum period. The microbiota of saliva, rumen fluid, and fecal samples were assessed using 16S rRNA sequencing. The rumen volatile fatty acid and blood parameters were also quantified.

**Results:**

The content of volatile fatty acids (VFAs) and blood parameters showed opposite tendency to each other and reached to stability at 2 M. Rumen microbiota showed the highest alpha diversity compared to other two sites. At phylum level, the oral cavity was dominated by Proteobacteria, while most dominant phylum in rumen and feces were Firmicutes and Bacteroidetes, respectively. The dominant genera in oral cavity were Moraxella and Bibersteinia, while genera Prevotella 1 and Ruminococcaceae UCG-005 were dominant in rumen and fecal samples, respectively.

**Discussion:**

Microbial network analysis revealed that most of the active genera in all networks belonged to phylum Firmicutes, indicating the importance of this phyla during postpartum period of lactating cattle. The functional analysis revealed distinct division of labor among three gastrointestinal sites associated with defense, fatty acid synthesis, and maintaining health of host. All in all, our findings provide insights into the metabolic and microbial changes of lactating Tibetan cattle and help to the improvement of the management strategies.

## 1 Introduction

Dairy cattle play significant role in world agriculture by providing milk and milk products. The postpartum period of dairy cattle is an important phase of their life mainly associated with the changes in physiology, rumen function, and energy metabolism. The postpartum period is characterized by negative energy balance due to transition from pregnancy to lactation and thereby linked with the increased risk of digestive and immune disorders (Vergara et al., 2014). Approximately 30–45% of postpartum cattle develop some type of clinical disease in the first 30–60 days of lactation (Edelhoff et al., 2020). Improper management of cattle during postpartum period results in the loss of productivity and fertility.

The diversity of gastrointestinal tract microbiota of the ruminants aid in the digestion of various indigestible feedstuffs into metabolites such as short chain fatty acids and amino acids which are utilized by the host animal for maintenance and growth. Many factors such as diet, age, genotype, and health status are known to alter the microbial composition of gut community in ruminants. The microbial communities of gastrointestinal tract of ruminants have been shown to influence the host health in term of metabolism, nutrient utilization, and immune responses. Moreover, they also impact the milk production by regulating feed intake.

Studies have shown that gut microbial composition undergoes drastic changes during the postpartum period. The dynamic shift in rumen microbiota from 1 week before and 1 week after postpartum in Holstein dairy cows was reported (Lima et al., 2015). Similarly, alterations in rumen bacterial communities from 3 weeks before and 3 weeks after parturition in response to the physiological and nutritional changes in dairy cow were observed (Zhu et al., 2017). Study on lactating cow from 1 to 14 days after calving revealed increased abundance of VFA producing genera in rumen (Huang et al., 2021). Prepartum and postpartum analysis of rumen microbiomes showed higher relative abundance of cellulolytic and amylolytic bacteria in dairy cow, respectively (Lima et al., 2015). Since there is a high demand for energy during the postpartum period of the cattle mainly due to milk production, and as the gut microbiota is closely associated with energy acquisition and metabolism, so variations in microbial community in different parts of digestive tract during postpartum period is of interest.

Given this paucity of data, it is clear that more work is required to better understand the influence of the gastrointestinal microbiota during the postpartum period. In particular, a deeper understanding of the freshening period (2 weeks after parturition) is necessary, as about 50% of all cows experience low dry matter intake (DMI) during this period, resulting in a state of negative energy balance (Wankhade et al., 2017). Increasing evidence showed that improving DMI of fresh cows alleviated the negative energy balance and increased the downstream milk production of dairy cows (Huang et al., 2021). Besides, the milk production of cows reaches its highest level in the 1st month of the postpartum period and the negative energy balance of cows usually alleviates after the 2nd month of the postpartum period.

The Tibetan cattle breed, which is indigenous to the high altitude regions of China, is known for its ability to adapt to harsh environmental conditions, including limited feed availability and low temperatures. However, little is known about the temporal variations in blood metabolites, ruminal fermentation, and digestive tract microbiota in postpartum Tibetan cattle. Moreover, most of the studies have focused on gut microbial variations and our understanding in other parts of gastrointestinal tract is limited. Therefore, the present study aimed to investigate the temporal variations in blood metabolites, ruminal fermentation, and microbial community of oral, rumen, and gut in lactating Tibetan cattle during the first 8 weeks of postpartum. The results of this study will provide insights into the metabolic and microbial changes that occur during this critical period in Tibetan cattle and may lead to the development of management strategies to improve their overall health and productivity.

## 2 Materials and methods

### 2.1 Animals and experimental design

In this study, a total of nine 5 years old female Tibetan cattle (C) from the Yangnuo Specialized Yak Breeding Cooperative (34°43′19.66”N, 102°28′49.51”E) at Xiahe county of Gannan Tibetan Autonomous Prefecture, Gansu Province, China. All the animals involved in this experiment were from the same herd and they all grazed together in an alpine meadow on the Qinghai Tibetan Plateau (QTP), where the average altitude is 3,300 m and the average annual temperature is 4°C. There were abundant natural alpine meadow herbage and water resource, and the animals freely drank water from the local river or the snow meltwater. All animals included in this study were healthy during our sampling period and received no recorded therapeutic or prophylactic antibiotic treatment. All the animals were grazed from 7 a.m. to 6 p.m., and the samples were collected before the morning grazing.

### 2.2 Sample collection

From June to August 2020, a total of nine female Tibetan cattle were used for sample collections in four different periods, including the 1st week (1 W), the 2nd week (2 W), the 1st month (1 M), and the 2nd month (2 M) of the postpartum period. Rumen fluid, fecal samples and oral cavity samples of these nine female Tibetan cattle were collected in each period. Thus, there were 36 rumen fluid, 36 fecal samples and 36 oral cavity samples were collected for this experiment. Rumen fluid (R: *n* = 36; average 50 mL/per animal) was obtained by inserting a stomach tube into the rumen, and discarded the first 200 ml fluid to reduce saliva contamination. At the same time, fecal samples (F: *n* = 36) were obtained from the rectum of the animals using sterile gloves and lubricants. Samples from the oral cavity (Oc: *n* = 36) were collected by swabbing the mouth with a sterile cotton swab (Universal Transport Medium for Bacterium, Beijing, China) and immediately placing them in the sampling tube containing the protective solution (1.5 mL). All samples were immediately placed in liquid nitrogen, transported to the laboratory and stored at −80°C until further analysis. Additionally, the blood samples (*n* = 36, average 10 mL/per animal) were collected from each animal by puncture of jugular vein into non-oxalate tubes (Supplementary Figure 1A).

### 2.3 Measurement of rumen volatile fatty acid and blood parameters

The cryopreserved rumen fluid samples were thawed at 4°C and thoroughly mixed by vertexing, centrifuged at 15,000 × *g* for 10 min at 4°C, and the supernatants were analyzed for the measurement of volatile fatty acids (VFAs), including acetic acid (ACE), propionic acid (PRO), isobutyric acid (IBUT), butyric acid (BUT), isovaleric acid (IVAL), and valeric acid (VAL) by the Agilent gas chromatography (7890A GC system Agilent Technologies Inc, Santa Clara, CA, USA; Erwin et al., 1961).

The blood was centrifuged at 3,500 rpm (15 min at 4°C) and the supernatant (serum) was collected and transferred into new tubes for the subsequent biochemical analyses of the concentrations of serum glucose (Glu), triacylglycerols (TG), total protein (TP), total bilirubin (TBil), calcium (Ca), phosphorus (P), iron (Fe), and magnesium (Mg) using reagent kits and the Mindray BS-240VET Automatic Hematology Analyzer (Mindray Corporation, Shenzhen, China).

### 2.4 DNA extraction and sequencing

Total genomic DNA from all the samples (*n* = 108) was extracted using hexadecyl trimethyl ammonium bromide (CTAB) method (Honoré-Bouakline et al., 2003). DNA concentration and purity were monitored using 1% agarose gels. DNA was diluted to a final concentration of 1 ng/μL using sterile distilled water. The 16S rRNA gene V4 region was PCRamplified using the specific primers: 515F 5′-GTGCCAGCMGCCGCGGTAA-3′ and 806R 5′-GGACTACHVGGGTWTCTAAT-3′ with barcodes (Caporaso et al., 2011). All PCR reactions were carried out in 30 μL reactions having 15 μL of Phusion^®^ HighFidelity PCR Master Mix (New England Biolabs), 0.2 μM of forward and reverse primers, and ~10 ng template DNA. PCR profile consisted of initial denaturation at 98°C for 1 min, followed by 30 cycles of denaturation at 98°C for 10 s, annealing at 50°C for 30 s, and elongation at 72°C for 30 s, and final extension at 72°C for 5 min. Amplicons were purified with Qiagen Gel Extraction Kit (Qiagen, Germany), sequencing libraries were generated using TruSeq^®^ DNA PCRFree Sample Preparation Kit (Illumina, USA) following manufacturer's recommendations and index codes were added. The library quality was assessed on the Qubit^@^ 2.0 Fluorometer (Thermo Scientific) and Agilent Bioanalyzer 2100 system. At last, the library was sequenced using Illumina NovaSeq platform and 250 bp pairedend reads (PE 250) were generated.

### 2.5 Sequencing data processing

All raw pairedend sequences were imported to QIIME2 pipeline (version 2020.8.0; Bolyen et al., 2019). Primers were removed using Cutadapt plugin (qiime cutadapt trimpaired -pminimumlength 200). The DADA2 plugin was used to generate denoised feature sequences (amplicon sequence variant, ASV) and feature table (qiime dada2 denoisepaired -ptrimleftf 15 -p- trimleftr 20 -ptrunclenf 0 -ptrunclenr 0 -pn-threads 6; Callahan et al., 2016). Feature sequences with frequency ≤ 4 were discarded. Reference sequences were extracted from SILVA database (release 132) using specific primers for 16S V4 region (qiime featureclassifier extractreads; Quast et al., 2013). The Naive Bayes classifier (qiime featureclassifier fitclassifiernaivebayes) was trained for taxonomic annotation (qiime featureclassifier classifysklearn). The ASVs assigned to mitochondria and chloroplast were excluded from feature table. PICRUST2 software (https://github.com/picrust/picrust2) was used to predict functional abundances of microbiota (Douglas et al., 2020).

### 2.6 Statistical analysis

Alpha diversity indices of microbiota (Shannon, observed ASVs and Pielou evenness) were calculated using “qiime diversity alpha.” Non-parametric Wilcoxon rank sum test was performed to compare alpha diversity between groups. Principle coordinate analysis (PCoA) plot was generated based on the Aitchison distance as recommended by Gloor et al. (2017). Permutational analysis of variance (PERMANOVA) and Analysis of similarities (ANOSIM) were applied to test group difference based on distance matrix using vegan package (Díaz-Sánchez et al., 2019). LefSe (Linear discriminant analysis Effect Size) and ANCOM (Analysis of composition of microbiomes) were both used to test the difference in taxa abundance and functional abundance (Segata et al., 2011; Mandal et al., 2015). The contribution of the ecological processes that determine community assembly in digestive tract was analyzed as described previously (Stegen et al., 2012, 2013). Interaction network was constructed using ASVs present in ≥50% of samples in oral cavity, feces and rumen, respectively. Briefly, (i) the ASV correlation was calculated using SparCC algorithm (Watts et al., 2019). (ii) The statistical significance of correlations was calculated from 1,000 bootstrap iterations. (iii) Network (*p* < 0.01 and |SparCC| ≥ 0.6) property calculation and module detection were employed using igraph package (Csardi and Nepusz, 2005). (iv) Network visualization was performed in Cytoscape 3.7.1 software. We partitioned the taxa into four groups based on node degree proportion or degree number (Supplementary Table 3): intramodule friendly taxa, intermodule friendly taxa, intramodule competitive taxa, and intermodule competitive taxa. Spearman's correlation analysis was used to identify correlations between taxa (taxon with average relative abundance <0.01% and frequency <0.5 was excluded), rumen volatile fatty acids (VFA) or blood biochemical parameters. Significant *p*-values were adjusted using the BenjaminiHochberg method. Code used for analyses is available at: https://github.com/wangpeng407/DTMicrobiota_Analysis.

## 3 Results

### 3.1 VFAs and blood parameters

The content of VFAs including ACE, PRO, IBUT, VAL, and IVAL increased from 1 to 2 W, and reached to stability after 2 W in the postpartum period. However, the content of BUT exhibited an increase from 1 to 2 W, then decreased from 2 W to 1 M and finally reached to stability after 1 M during the postpartum period (Supplementary Figure 1B). The blood parameters including Glu, TG, TP, TBil, Ca, and P showed a significantly decreasing tendency before 1 M, while Mg showed increasing tendency before 2 M, then achieved a stability. The content of Fe remained stable during the postpartum period (Supplementary Figure 1C).

### 3.2 Sequencing depth and coverage

After quality filtering, a total of 7,636,651 high quality reads were obtained from 108 samples with an average of 70,710 ± 9,752 (mean ± SD) per sample. We obtained 18,242 ASVs after denoising, merging and removing the chimeras. Observed species curves for all samples showed the adequate sequencing depth were achieved suggesting the coverage of major members of bacterial community.

### 3.3 Microbial diversity analysis

**Figure 1:**
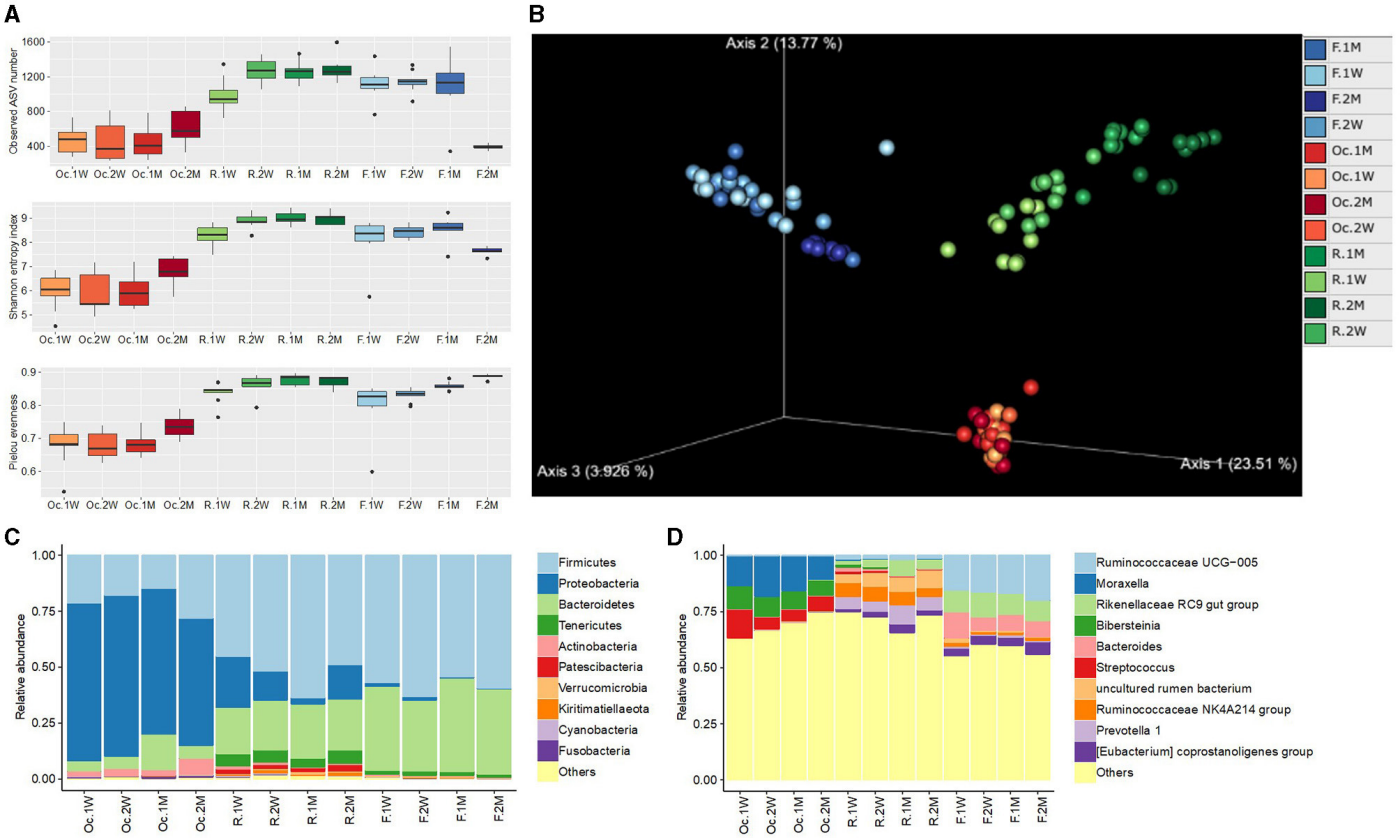
Boxplot of alpha diversity indices in different sites of the digestive tract in postpartum cattle **(A)**. PCoA plot of the digestive tract microbiota based on Aitchison distance **(B)**. Relative abundance of microbiota at phylum **(C)** and genus **(D)** levels. Oc, R, and F represent oral cavity, rumen, and feces, respectively. 1 W, 2 W, 1 M, and 2 M represent weeks 1, 2, months 1, 2, respectively.

Among the three sites in digestive tract, rumen microbiota showed the highest alpha diversity (richness and evenness) compared to other two sites. Oral cavity displayed the lowest alpha diversity which decreased from 1 W until 1 M, however, dramatically increased at 2 M. In rumen, it increased with time and reached to stability at 1 M. Fecal microbial diversity showed the stability from 1 W to 1 M and dramatically decreased during 2 M (Figure 1A, Supplementary Table 2).

Beta diversity analysis displayed distinct difference in microbial community among the three sites. Oral cavity microbiota exhibited random change at different time points. The rumen and fecal microbiota showed a regular change with the passage of time and became convergent at 2 M (Figure 1B).

### 3.4 Taxonomic analysis

At phylum level, the oral cavity was dominated by Proteobacteria (66.1%), Firmicutes (20.5%), Bacteroidetes (7.8%), and Actinobacteria (3.8%; Figure 1C). The most dominant phylum in rumen were Firmicutes (52.3%), Bacteroidetes (22.4%), Proteobacteria (13.5%), Tenericutes (5.2%), and Patescibacteria (2.0%). While, Firmicutes (58.5%), Bacteroidetes (37.2%), Tenericutes (1.8%), and Proteobacteria (1.0%) were predominant in fecal samples.

The dominant genera in oral cavity were *Moraxella* (14.4%), *Bibersteinia* (11.5%), and *Streptococcus* (8.4%). Genera *Prevotella 1* (6.2%), *uncultured rumen bacterium* (6.1%), *Ruminococcaceae NK4A214 group* (5.6%), and *Ruminococcaceae UCG-014* (4.2%) were abundant in rumen. Genera *Ruminococcaceae UCG-005* (17.1%), *Rikenellaceae RC9* gut group (9.8%), *Bacteroides* (7.9%), and *Eubacterium coprostanoligenes* group (4.2%) were dominant in fecal samples (Figure 1D).

### 3.5 Formatting of mathematical components

**Figure 2:**
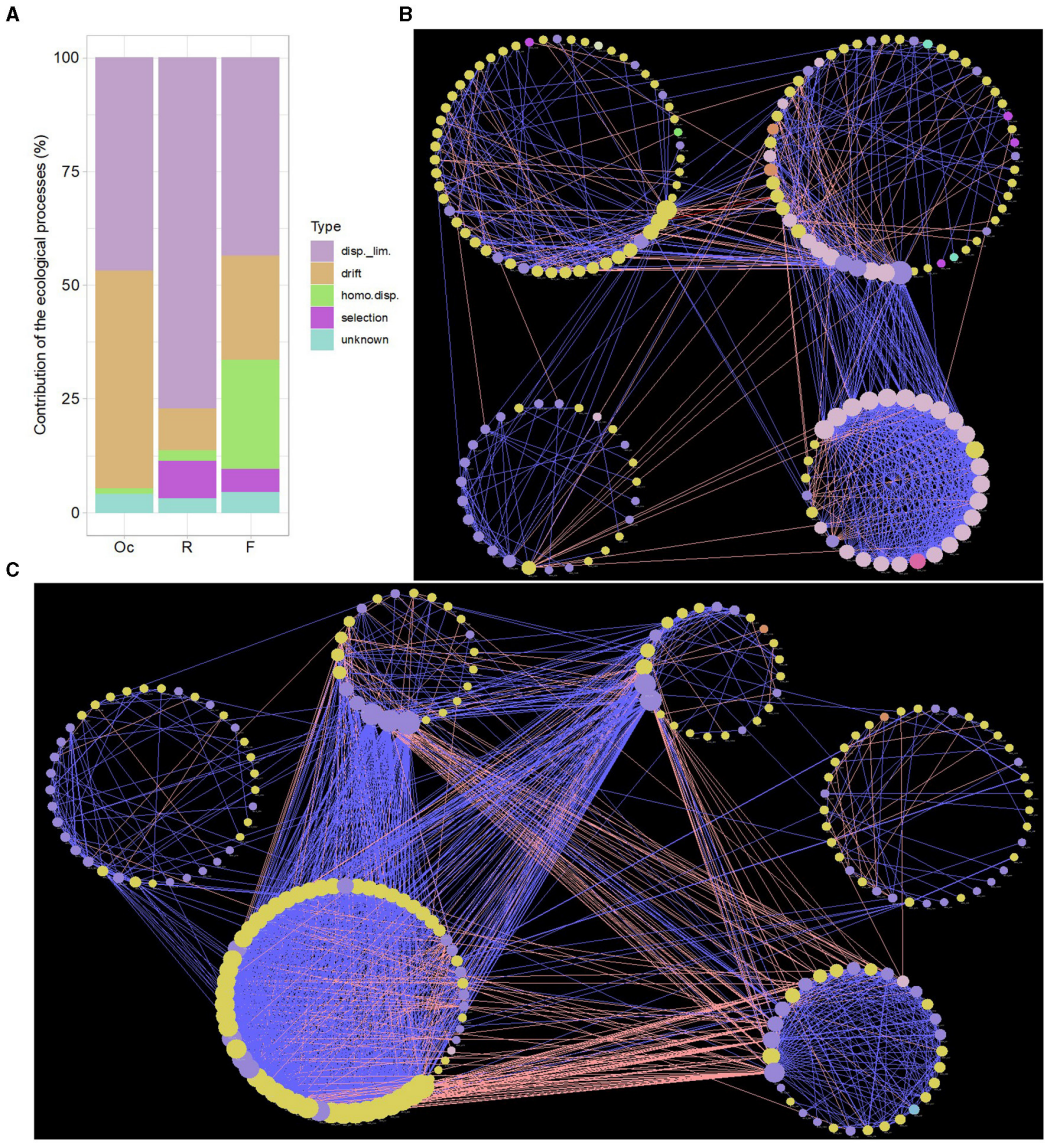
Contribution of the ecological processes that determine community assembly in oral cavity, rumen and feces. “disp._lim.” and “homo.disp.” represent dispersal limitation and homogenizing dispersal, respectively **(A)**. Microbial co-occurrence network of ASVs in rumen **(B)** and feces **(C)** based on SparCC algorithm. Nodes correspond to ASVs and edges to the correlation. Node size is proportional to the degree number. Node color represents the associated phylum for each ASV. Edge width displays the strength of correlation. Blue edge indicates positive correlation, pink negative. Each large circle represents a module detected by Louvain method.

ASV abundance and phylogenetic tree were used to quantify the relative contribution of ecological processes that determine the community turnover (Figure 2A). We found that processes regulating community assembly differ greatly among different sites in the digestive tract microbiota, but dispersal limitation displayed a strong signal influencing the community turnover patterns in all sites (oral cavity: 46.9%, rumen: 77.1%, feces: 43.7%). Drift was also a main process that influence the community turnover of microbiota in oral cavity (47.5%), highlighting the random changes of microbiota abundance in this site, followed by fecal (22.5%) and rumen (9.0%) microbiota. Selection played a certain role in rumen (8.5%), suggesting the rumen microbial community structure was also affected by nichebased process due to fitness differences. Homogenizing dispersal processes was also very important for governing the community patterns of fecal microbiota (24.1%), showing increased dispersal rates in this site.

### 3.6 Microbial interaction

The ASVs distributed in at least 50% of samples were chosen to perform co-occurrence network. The microbial interaction pattern was greatly distinct in different sites of the digestive tract of cattle. The microbial network in fecal samples showed the highest transitivity (0.636), followed by rumen (0.555) and oral cavity microbiota network (0.340), showing the property of “small world” in microbiota of rumen and feces. The number of total degree also supported this finding (Supplementary Table 3).

We used Louvain method (Supplementary Table 4) to partition microbial network into clusters to identify key microbial taxa involved in maintaining community structure. We selected the clusters showing more than 20 nodes. In case of oral cavity, the co-occurrence pattern of microbial network was very sparse, showing a random and instable community structure (Supplementary Figure 5). We identified four clusters in in rumen microbial network (Figure 2B). RM3 cluster was moderately connected with 57 nodes and most of them were assigned to Firmicutes. RM1 cluster was similar to RM3 cluster, containing 57 nodes and two of them were intercluster positively interacting taxa (Supplementary Table 3). The nodes having more than 20 connections (degree > 20) were mainly mapped to Proteobacteria and Bacteroidetes. RM2 cluster showed more positively connected taxa and most of the nodes showing positive interaction were assigned to Proteobacteria. There was also a strong positive interaction between RM1 and RM2 clusters, showing that RM1 cluster or Proteobacteria in this cluster paly key role in regulating the microbiota network in rumen. The fecal microbial network contained six clusters with high degree nodes compared to rumen (rumen: 14, feces: 55; Figure 2C, Supplementary Table 3). The tightly connected FM2 cluster, mainly made up of Firmicutes, had a highest number of positively and negatively interacting taxa. These taxa showed strong interaction with FM1 (high degree nodes dominated by Bacteroidetes) and FM4 (high degree nodes dominated by Bacteroidetes) clusters. FM3 cluster (mostly assigned to Bacteroidetes) was identified as a competitive cluster, as it contained high number of negatively interacting taxa with FM1, FM4, and FM2 clusters. These results demonstrated that the microbiota network in feces was mainly regulated and balanced by Firmicutes (Ruminococcaceae) in FM2 cluter and Bacteroidetes in FM1, FM4, and FM3 clusters.

### 3.7 Association between blood parameters rumen VFA and microbial community

The envfit results showed that total protein (TP), phosphorus ion (P), and total bilirubin (T.bil) strongly and significantly correlate with rumen microbiota (*p*-value ≤ 0.001, *R*^2^ > 0.35). Calcium ion (Ca), triglyceride (TG), and valeric acid (VAL) showed moderate and significant connection (Supplementary Table 5).

**Figure 3:**
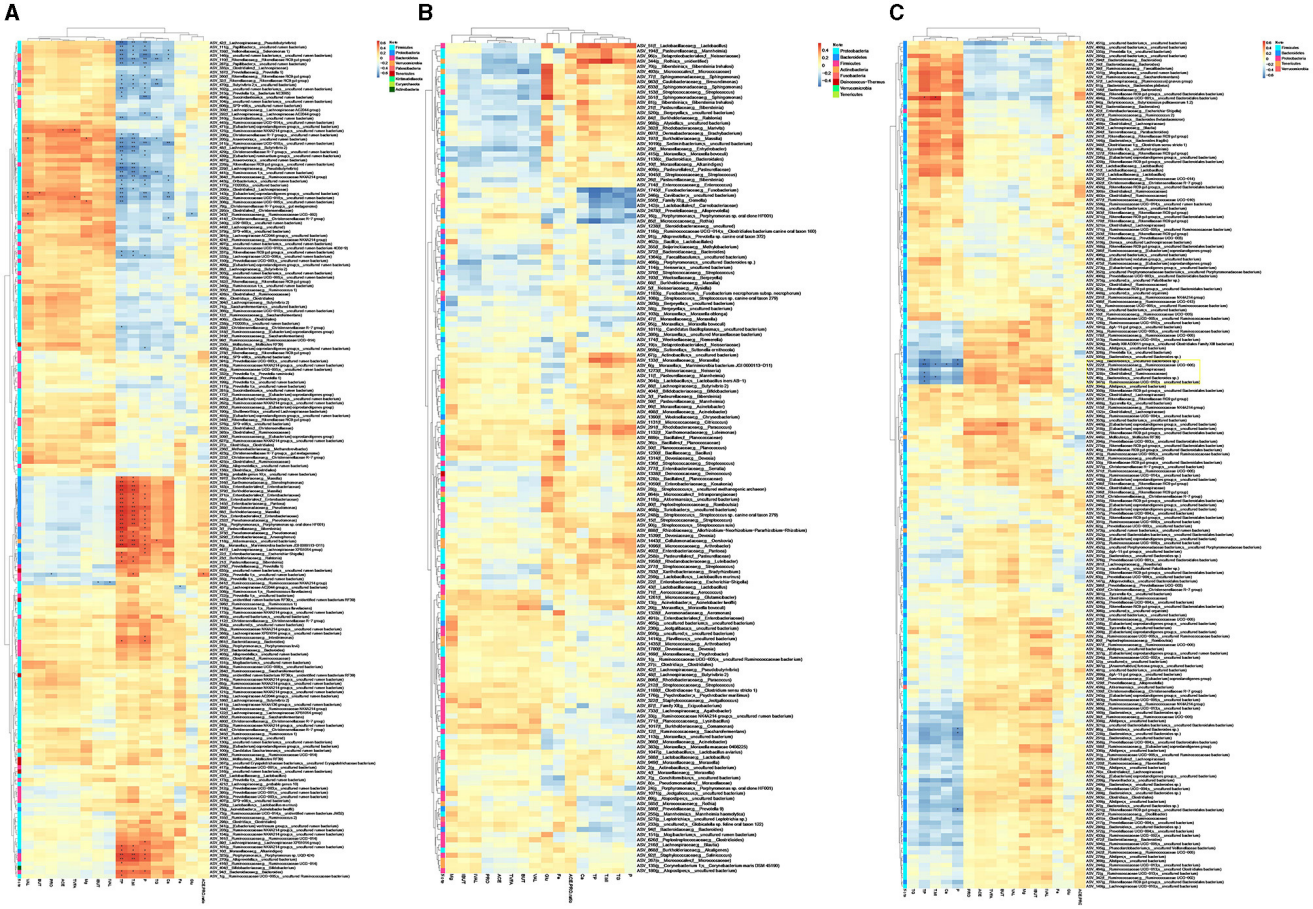
Heatmap of correlations between ASVs and VFAs or blood biochemical parameters in oral cavity **(A)**, rumen **(B)**, and feces **(C)**. ACE, acetic acid; PRO, propionic acid; IBUT, isobutyric acid; BUT, butyric acid; IVAL, isovaleric acid; VAL, valeric acid; TVFA, total volatile fatty acid; ACE.PRO.ratio, ratio of acetic acid to propionic acid; Glu, glucose; TG, triglyceride; TP, total protein; Tbil, total bilirubin; Ca, calcium ion; P, phosphorus ion; Fe, ferric ion; Mg, magnesium ion. * and ** indicate *p* < 0.05 and *p* < 0.01, respectively.

Correlation analysis showed rumen microbiota that strongly connected with host blood parameters and rumen VFAs (Figure 3B), while the microbiota in oral cavity and feces showed no or little effects (Figures 3A, C). The ASVs correlated with host parameters were separated into four clusters (Supplementary Table 6). Cluster I (mainly assigned to Firmicutes and Bacteroidetes belonged to the module of RM1, RM3, and RM7) and Cluster II (Firmicutes in RM1, RM2, RM3, and RM7) were negatively correlated with TP, T.bil, P, TG, and Ca. While Cluster III (Proteobacteria in RM1 and RM2) and Cluster IV (Firmicutes and Bacteroidetes in RM1) were positively correlated with TP, T.bil, and P. Interestingly, although we observed little associations between ASVs and VFAs, there was still an outcome that was found in Cluster II and Cluster III. Cluster II species may contribute to the production of VFAs based on the positive correlation. There was a negative correlation between Cluster III species and VFAs. Rumen microbiota may play more important roles than oral cavity and fecal microbiota in host metabolism and nutrition absorption. Furthermore, rumen microbiota had a stronger effect on protein, phosphorus ion and bilirubin than volatile fatty acids.

### 3.8 Functional profiles

**Figure 4:**
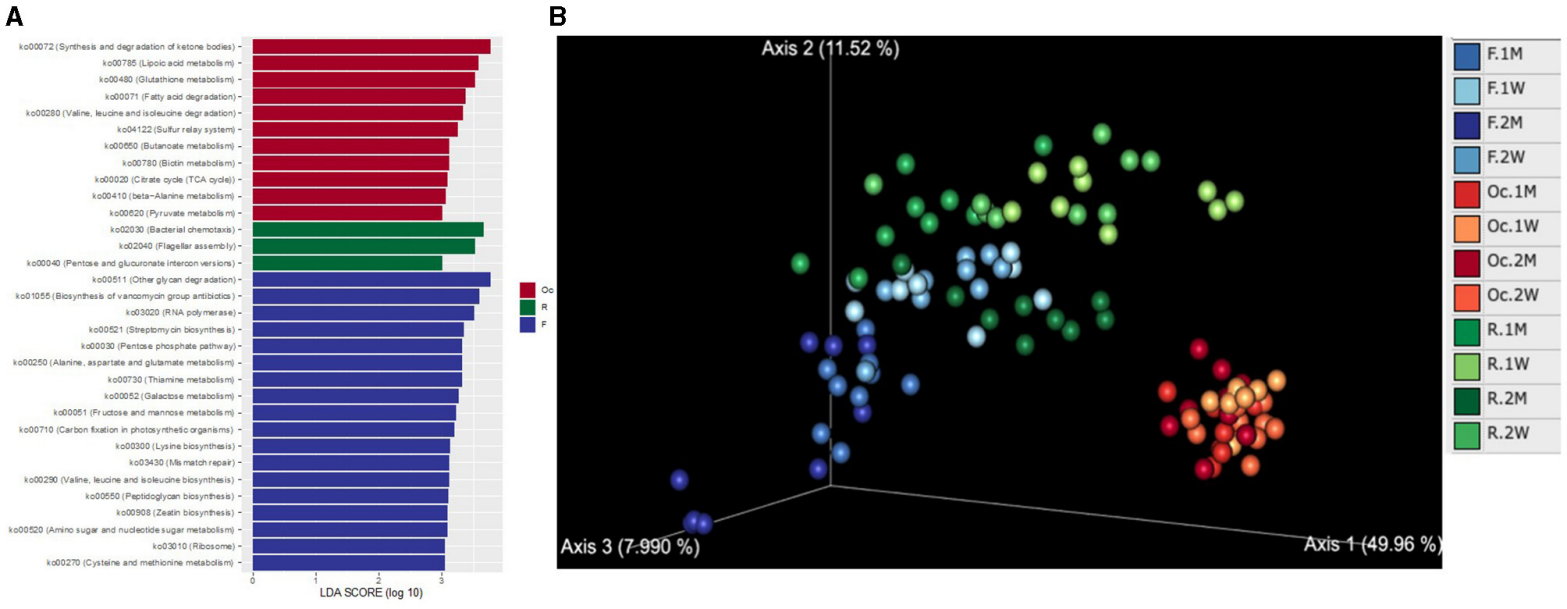
PCoA plot of KEGG KO abundance predicted by PICRUST2 based on Aitchison distance **(A)**. LEfSe and ANCOM highlight differential KEGG pathways among different sites of the digestive tract in postpartum cattle **(B)**. LDA score distribution of KEGG pathway in different sites of the digestive tract at weeks 1, 2, months 1, 2 in postpartum cattle. Only consider KEGG pathways with LDA score ≥ 3 and average abundance ≥ 0.5%.

To investigate the KO patterns across different sites in the digestive tract, we performed PCoA of KO abundance predicted by PICRUST2. Consistent with the variation in microbiota, the microbial function profile also possesses the stratification across different sites and different time (Figure 4A). The microbial function analysis showed a large turnover across time in postpartum period especially in rumen and feces. Differential KEGG pathway analysis showed that the microbiota in the three sites have different functionality (Figure 4B). The microbial function in oral cavity was dominated by synthesis and degradation of ketone bodies (ko00072), lipoic acid metabolism (ko00785), geraniol degradation (ko00281), and so on. While in rumen, it was bacterial chemotaxis (ko02030), flagellar assembly (ko02040), and Pentose and glucuronate interconversions (ko00040). The pathways of other glycan degradation (ko00511), biosynthesis of vancomycin group antibiotics (ko01055), and RNA polymerase (ko03020) were dominated in the fecal microbiota.

## 4 Discussion

The gastrointestinal tract of cattle contains a diverse microbial ecosystem involved in regulating host metabolism and health and even a small physiological change drastically impacts the community structure of gut microbiota. Cattle undergo various physiological changes during postpartum period to adapt to lactating state and maintain health. Numerous studies have reported that gut microbiota is closely related to the lactation of cattle (Monteiro et al., 2022). In this study, we explored the temporal variations in oral, rumen, and fecal microbiota in postpartum Tibetan cattle with the aims to understand the patterns of composition and functionality of microbiome with the progress of postpartum period.

The metabolism of lipid, glucose, protein, and minerals in the body of dairy cows undergo significant changes especially during the transition period in order to adjust to the needs of lactation and maintain their body functions (Green et al., 2012). In our study, we recorded increased production of SCFA during first 2 weeks which reached to stability after 1st month. The large quantities of SCFAs perform important role in supporting high milk production by providing metabolizable energy (Tokach et al., 2019). We assume that high absorption of VFA from rumen epithelium to meet the energy demand for synthesis of colostrum and milk and cope with physiological changes causes decrease amount of VFA in rumen. However, they started to increase during 2nd week was to fulfill the high energy requirements of lactating cattle after recovering from immediate postpartum changes. During postpartum period, nutrients and minerals required for milk production such as glucose, triglycerides, proteins, Ca, P, etc. are transferred to the mammary gland through blood, which explain their high amount during first 2 weeks in this study (Svennersten-Sjaunja and Olsson, 2005).

Gut microbiota plays vital role in maintaining normal functioning of body and is modulated by stress and physiological changes (Hasan and Yang, 2019). Studies have shown that gut microbiota is associated with maternal health and recovery from pregnancy stress (Tröscher-Mußotter et al., 2022; Gu et al., 2023). We observed increase in oral microbial diversity with the progress of postpartum period which might be associated with licking of young calves and protection from pathogenic colonization or increased foraging after early lactation (Jouany, 2006; Barboza-Solís and Acuña-Amador, 2021). Rumen microbiota are linked with the production of SCFA and microbial protein by degrading plant lignocellulosic material, which explain their high diversity during early stage of postpartum compared to fecal samples (Abecia et al., 2018). Overall, an increase in nutritional requirements and metabolism level during postpartum due to lactation might be responsible for the increase in diversity.

We observed significant microbial variations in the various sites of gastrointestinal tract of postpartum cattle. Oral cavity was dominated by *Moraxella, Bibersteinia*, and *Streptococcus* which are known to play important role in oral health. Similar results were also observed in the studies on dairy calves and human (Barden et al., 2020; Liu et al., 2021). Genera *Prevotella* and *Ruminococcaceae* are reported to play vital role in biohydrogenation process which is important for the flow of saturated fatty acids from the rumen to incorporate in milk fat, thus explains their abundance in rumen (Huws et al., 2011; Dewhurst and Moloney, 2013). Both genera also showed higher interactions in rumen microbial network which further highlight their importance in the rumen of lactating cattle. We also observed higher abundance of genus *Ruminococcaceae* in fecal samples was also very active in the fecal microbial network.

The genus *Rikenellaceae RC9* gut group, found in fecal matter of animals and human, can utilize crude fiber as carbohydrate source and generate energy in the form of acetate and propionate (Graf, 2014). High abundance of this genus was reported in the most efficient steers and shows its association with health of animal (Welch et al., 2021). In this study, *Rikenellaceae RC9* gut group was highly abundant in fecal matter which might be linked to their role in the maintaining the health of lactating cattle as revealed by positive correlation with SCFA. Genus *Bacteroides* degrades polysaccharides, produces anti-inflammatory molecules, and is involved in the maintenance of gut health (Brown et al., 2019). We observed higher abundance of this genus during 1st week of postpartum which might be involved in maintaining the oxidative balance induced due to delivery and maintaining health of lactating cattle. The higher abundance of this genus was also reported in healthy individuals compared to the diseased ones (Zhou and Zhi, 2016). We assumed that all these genera might be performing their role at their respective sites to maintain the health of the animal and to reduce the postpartum stress.

Microbiota perform synergistic functions to degrade plant fibers and to maintain metabolic health. Network analysis helps to dig deep in the interactions of microbial community and identifying the key taxa. The microbial interaction analysis in this study revealed that genera from phylum Firmicutes were highly active in gastrointestinal tract. Phylum Firmicutes mostly consist of diverse cellulolytic and fibrolytic bacterial genera (Zhang et al., 2021). Most of the active genera in rumen network belonged to phylum Firmicutes indicating the importance of this phyla during postpartum period of lactating cattle. There were not many interactions observed in the oral microbial network which might be linked to the instable structure of oral community. However, rumen and fecal microbial networks displayed complex and clear clustering of microbiota. In rumen, genera *Papillibacter* and *Anaerovorax* were the most active with maximum number of interactions in the network. Genus *Papillibacter* is known to reduce the pH of rumen by producing metabolites to cause the death of pathogenic bacteria, thus aids in maintenance of healthy rumen environment (Ahmad et al., 2022). Genus *Anaerovorax*, a strict anaerobe, is known to produce acetate and butyrate thus might be involved in providing energy to lactating cattle in this study (Matthies et al., 2000). Genus *Oscillibacter* showed higher interactions in fecal microbial network which produces anti-inflammatory metabolites and is involved in the maintenance of gut barrier integrity (Lam et al., 2012). The results of this study indicates that microbial community plays important role in the survival and stability of Tibetan cattle during the postpartum period by efficiently digesting feed to generate VFA and combating pathogens to maintain the health of cattle. This study provides a baseline for managing cattle in various environmental conditions by suggesting microbial manipulation an efficient strategy to maintain health and survival of cattle during the postpartum period.

## 5 Conclusions

The present study offers new insight into the role of the gastrointestinal bacterial community in the lactating cattle during postpartum period. We recorded functional specific bacteria in different parts of gastrointestinal tract. Oral microbiota was the most dynamic and dominated with oral health related bacteria. Rumen microbiota was abundant with fatty acid producing bacteria to aid in milk production, while fecal microbiota was associated with fiber degradation, energy generation, and maintenance of gut health. The network analysis revealed that genera belonging to phylum Firmicutes were highly active throughout the studied sites. This study will help in understanding how the variation in microbial community play role in reducing stress and maintaining health of cattle during postpartum period. Moreover, it will also provide theoretical and practical scientific basis to manage lactating cattle through manipulations of gut microbiota.

## Data Availability

The datasets presented in this study can be found in online repositories. The names of the repository/repositories and accession number(s) can be found at: https://www.ncbi.nlm.nih.gov/, PRJNA1051369.
