# Supplementary material for: Modulations in gastrointestinal microbiota during postpartum period fulfill energy requirements and maintain health of lactating Tibetan cattle
Source: Front Microbiol. 2024 Aug 20;15:1369173. doi: 10.3389/fmicb.2024.1369173 (PMC11368858; doi:10.3389/fmicb.2024.1369173)
Supplement: Supplementary file 1 [file Table_1.DOCX]

Supplementaty materials

Fig S1. Indication of sample collection in this study (A). Changes in volatile fatty acids (B) and blood parameters (C) in postpartum Tibetan cattle.

Fig S2. PCoA plot of the microbiota in oral cavity (n=36), rumen (n=36) and feces (n=36) based on Aitchison distance

Fig S3. Relative abundance of the microbiota at family level in oral cavity (n=36), rumen (n=36) and feces (n=36).


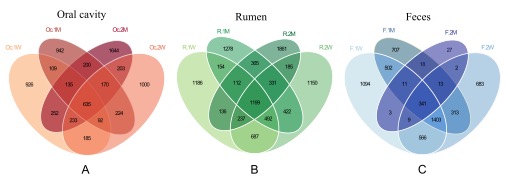


Fig S4. Venn diagram displaying shared and specific ASVs in oral cavity (n=36), rumen (n=36) and feces (n=36).

Fig S5. ASVs identified as biomarkers using Ancom, Lefse, and both in oral cavity (n=36), rumen (n=36) and feces (n=36).

Fig S6. Microbial co-occurrence network of ASVs in oral cavity based on SparCC algorithm. Nodes correspond to ASVs and edges to the correlation. Node size is proportional to the degree number. Node color represents the associated phylum for each ASV. Edge width displays the strength of correlation. Blue edge indicates positive correlation, pink negative. Each large circle represents a module detected by Louvain method.

Fig S7. Significantly abundant KO pathways during different time periods at three different sites.
